# BAM1/2 receptor kinase signaling drives CLE peptide-mediated formative cell divisions in *Arabidopsis* roots

**DOI:** 10.1073/pnas.2018565117

**Published:** 2020-12-07

**Authors:** Ashley D. Crook, Andrew C. Willoughby, Ora Hazak, Satohiro Okuda, Kylie R. VanDerMolen, Cara L. Soyars, Pietro Cattaneo, Natalie M. Clark, Rosangela Sozzani, Michael Hothorn, Christian S. Hardtke, Zachary L. Nimchuk

**Affiliations:** ^a^Department of Biology, University of North Carolina at Chapel Hill, Chapel Hill, NC 27599;; ^b^Department of Plant Molecular Biology, University of Lausanne, CH-1015, Lausanne, Switzerland;; ^c^Structural Plant Biology Laboratory, Department of Botany and Plant Biology, University of Geneva, CH-1211 Geneva, Switzerland;; ^d^Department of Plant and Microbial Biology, North Carolina State University, Raleigh, NC 27607;; ^e^Curriculum in Genetics and Molecular Biology, University of North Carolina at Chapel Hill, Chapel Hill, NC 27599

**Keywords:** *Arabidopsis*, receptor kinase, cell cycle, SHORT-ROOT, CLE peptide

## Abstract

Cell division is often regulated by extracellular signaling networks to ensure correct patterning during development. In *Arabidopsis*, the SHORT-ROOT (SHR)/SCARECROW (SCR) transcription factor dimer activates *CYCLIND6*;*1* (*CYCD6;1*) to drive formative divisions during root ground tissue development. Here, we show plasma-membrane-localized BARELY ANY MERISTEM1/2 (BAM1/2) family receptor kinases are required for *SHR*-dependent formative divisions and *CYCD6;1* expression, but not *SHR*-dependent ground tissue specification. Root-enriched CLE ligands bind the BAM1 extracellular domain and are necessary and sufficient to activate *SHR*-mediated divisions and *CYCD6;1* expression. Correspondingly, BAM-CLE signaling contributes to the restriction of formative divisions to the distal root region. Additionally, genetic analysis reveals that BAM-CLE and SHR converge to regulate additional cell divisions outside of the ground tissues. Our work identifies an extracellular signaling pathway regulating formative root divisions and provides a framework to explore this pathway in patterning and evolution.

## Significance

Proper elaboration of the plant body plan requires that cell division patterns are coordinated during development in complex tissues. Activation of cell cycle machinery is critical for this process, but it is not clear how or if this links to cell-to-cell communication networks that are important during development. Here we show that key cell divisions that generate the plant root are controlled by cell-to-cell signaling peptides which act through plant-specific receptor kinases to control expression of a specific *cyclinD* cell cycle regulatory gene. We show that *cyclinD* gene expression depends on both receptor signaling and the SHORT-ROOT transcription factor to ensure timely and robust cell division patterns.

**Fig. 1.**
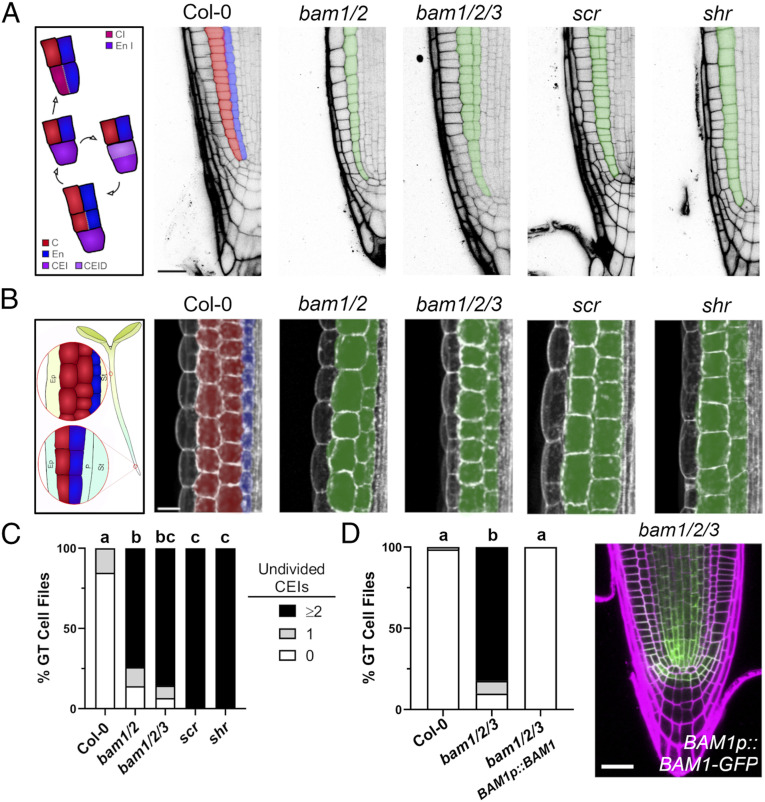
BAM1/2 receptor kinases are required for formative ground tissue divisions. Confocal images of *scr*, *shr*, and higher order *bam* mutants at 7 dag with similar defects in ground tissues in root (*A*) and hypocotyl tissues (*B*). Cortex (red), endodermis (blue), mutant ground tissue layers (green). (*C*) Undivided CEIs (0, 1, or ≥2) were quantified in each ground tissue (GT) cell file in each mutant (*n* = 88, Col-0; *n* = 54, *bam1/2*; *n* = 59, *bam1/2/3*; *n* = 139, *scr*; and *n* = 108, *shr*). (*D*) *BAM1p::BAM1-GFP* rescues CEI divisions in *bam1/2/3* mutant roots (*n* = 68, Col-0; *n* = 70, *bam1/2/3*; and *n* = 102, *bam1/2/3 BAM1p::BAM1-GFP*). Distributions were compared using a Kruskal–Wallis nonparametric test. C, cortex; En, endodermis; CEI/CEID, cortex/endodermal initial/daughter; CI, cortex initial; En I, endodermal initial; Ep, epidermis; P, pericycle; St, stele.

Correct patterning requires that cell division and differentiation are often coordinated among cells in developing tissues. Extracellular ligand-mediated signaling pathways contribute to this process, and in animals often directly regulate cell cycle progression (1). Plant development is controlled by diverse extracellular inputs; however, few clear connections between these signaling networks and the cell cycle machinery exist. *Arabidopsis* roots contain two internal ground tissue layers, the endodermis and cortex, generated postembryonically by formative divisions in cortex endodermal initial (CEI) cells and their CEI daughter (CEID) cells (2) (Fig. 1*A*). The SHORT-ROOT (SHR)/SCARECROW (SCR) transcription factor dimer promotes these formative divisions (3–5). SHR synthesized in the stele traffics into CEIs (6), endodermis, and quiescent center (QC) cells, where it activates *SCR* expression (7). Nuclear SCR/SHR complexes then directly activate *CYCLIND6*;*1* (*CYCD6;1*) transcription to promote CEID division (8). In contrast, SHR/SCR suppresses *CYCD6;1*-mediated middle cortex cell layer formation during root maturation (9). The *BARELY ANY MERISTEM* (*BAM*) receptor kinase subclade includes *BAM1-3* and *CLAVATA1* (*CLV1*) (10), with the highly similar *BAM1* and *BAM2* acting redundantly in male germline development (11) and additively with *BAM3* and *CLV1* in shoot stem cell regulation (12). While *BAM3* is involved in phloem differentiation (13), the function of *BAM* receptors in root patterning is largely unknown. Here, we demonstrate that plasma membrane-associated BAM1/2 receptor kinases, and a subset of the 32-member CLE family peptide ligands (14), are critical regulators of formative root cell divisions and modulate *SHR*-dependent *CYCD6;1* expression.

## Results

Examination of 7-d-old *bam1/2/3* triple and *bam1/2* double mutant plants revealed a lack of formative divisions in presumptive CEI/CEID cells resulting in the generation of a single ground tissue layer as in *shr* and *scr* mutants (Fig. 1 *A* and *C* and *SI Appendix*, Fig. S1 *A* and *B*), with *bam1* single mutants displaying a quantitative delay in CEI divisions in 5-d-old seedlings (*SI Appendix*, Fig. S1*A*). This single ground tissue layer occasionally divided forming presumptive ectopic middle cortex cells as previously noted in *scr* and *shr/+* mutants (2, 15), with divisions nearest to CEIs being rarer, but the presence of two contiguous cell layers were absent in all cases in *bam* mutant plants. *bam1/2/3* and *bam1/2* plants also displayed reduced ground tissue layers in hypocotyls, phenocopying *shr* and *scr* mutants (16) (Fig. 1*B*). As previously reported, *BAM1* and *BAM2* are expressed broadly in the stem cell niche including CEI and CEID cells for *BAM1*, with *BAM3* being primarily in the developing phloem lineage and pericycle (17, 18) (Fig. 1*D* and *SI Appendix*, Fig. S1 *C* and *D*). Consistent with this, expression of a functional *BAM1-2xGFP* fusion from the native *BAM1* promoter fully restored CEI divisions and ground tissue layer number in *bam1/2/3* triple mutant plants (17) (Fig. 1*D* and *SI Appendix*, Fig. S1*C*). These results demonstrate that *BAM1/2* regulate the formative cell divisions which give rise to root and hypocotyl ground tissues.

**Fig. 2.**
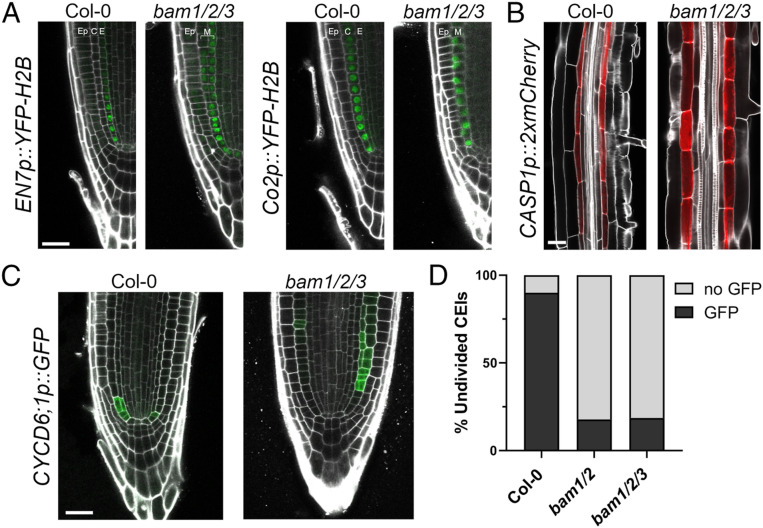
BAM1/2 are specifically required for *CYCD6;1* activation during formative divisions. (*A*) *EN7p::YFP-H2B* and *Co2p::YFP-H2B* are both expressed in the *bam1/2/3* single mutant layer (7 dag). (*B*) *CASP1p::2xmCherry*, a marker of the Casparian strip, is expressed in *bam1/2/3* (4 dag). (*C*) Representative images of Col-0 and *bam1/2/3* expressing *CYCLIND6;1p::GFP* (3 dag). (*D*) Undivided CEI cells directly adjacent to the QC with or without GFP signal were quantified in Col-0, *bam1/2*, and *bam1/2/3* at 3 dag (with GFP, *n* = 54/60, Col-0; *n* = 26/146, *bam1/2*; *n* = 18/97, *bam1/2/3*). Ep, epidermis; C, cortex; E, endodermis. (Scale bar, 25 µm in *A*–*C*.)

We next examined the identity of the mutant ground tissue layer in *bam1/2/3* mutant plants. *scr* and *shr* mutants both have a single ground tissue layer, expressing mixed cortex/endodermis and cortex identity, respectively (5). Like *scr* mutants, the single ground cell layer in *bam1/2/3* mutants expressed both cortex (*Co2*) and endodermal (*EN7*) reporter gene expression (Fig. 2*A*) and expressed *CASP1* (19, 20) (endodermal differentiation, Fig. 2*B*), indicating that SHR-mediated endodermal specification and differentiation was not impaired in *bam1/2/3* triple mutants. Consistent with this, SHR was expressed in the stele of *bam1/2/3* triple mutants, moved into the mutant ground tissue CEIs, where it was retained and localized to the nucleus, and activated *SCR* expression as in wild-type plants (*SI Appendix*, Fig. S2 *A*–*C*). As such, BAM1/2 are not required for SHR trafficking (2), SHR sequestration (21, 22), or SHR endodermal target gene expression. *BAM1/2* do not respond to *SHR* perturbation and are not direct SHR/SCR targets (7, 8, 23, 24). We next asked if BAM1/2 signaling impacted SCR/SHR-mediated *CYCD6;1* activation by imaging the expression of a transcriptional *CYCD6;1* reporter in *bam1/2* and *bam/1/2/3* mutant roots (8). In 3-d-old wild-type plants, *CYCD6;1* expression precedes CEID divisions (8). Strikingly, in 3-d-old *bam1/2* and *bam1/2/3* mutant seedlings, *CYCD6;1* expression was rarely observed in undivided CEIs (Fig. 2 *C* and *D*). As previously noted in *shr/+* and *scr* mutants (2), ectopic divisions in 3-d-old *bam1/2/3* mutant seedlings were also associated with proximal *CYCD6;1* expression (Fig. 2*C*). Collectively, these data show that *BAM1/2* are not just necessary for formative CEI divisions but also for the correct expression of the SHR/SCR target gene *CYCD6;1*.

**Fig. 3.**
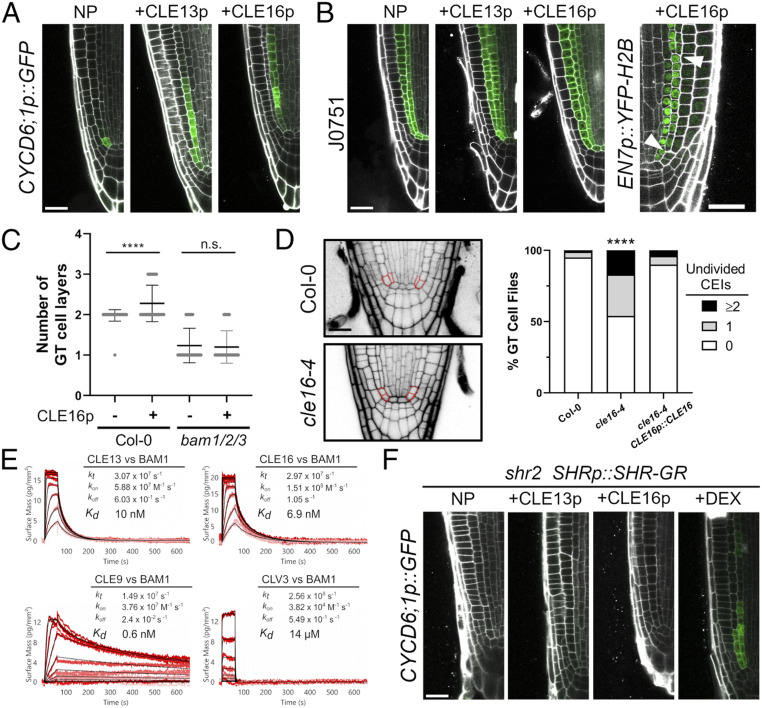
BAM-CLE signaling regulates SHR-dependent cell division. Expression of *CYCLIND6;1* (*A*) and J0571 (*B*) in Col-0 roots with no peptide (NP), CLE13p, or CLE16p treatments. Expanded *CYCLIND6;1p::GFP* expression in Col-0 was observed when treated with CLE13p (*n* = 6/8) and CLE16p (*n* = 28/33) compared to no peptide controls (*n* = 0/23). (*B*) The *EN7* marker becomes restricted to innermost cells following ectopic CLEp-induced asymmetric divisions (white arrowheads). (*C*) Col-0 roots show an increase in the number of ground tissue layers while *bam1/2/3* are not affected by CLE16p treatment [*n* = 50, Col-0(−); *n* = 54, Col-0(+); *n* = 64, *bam1/2/3*(−); and *n* = 65, *bam1/2/3*(+)]. Distributions were compared using a Mann–Whitney nonparametric *t* test (****P ≤ 0.0001). (*D*) CEI division defects in the *cle16-4* mutant are fully restored with *CLE16p::CLE16* (*n* = 127, Col-0; *n* = 118, *cle16-4*; and *n* = 152, *cle16-4 CLE16p::CLE16*). Distributions were compared using a Kruskal–Wallace nonparametric test (****P ≤ 0.0001). (*E*) Quantitative binding kinetics of CLE peptides versus the BAM1 ectodomain by GCI. Shown are sensorgrams with raw data in red and their respective fits in black. Binding kinetics were analyzed by a one-to-one binding model with mass transport. Table summaries of kinetic parameters are shown: k_t_, mass transport coefficient; k_on_, association rate constant; k_off_, dissociation rate constant; and K_d_, dissociation constant. (*F*) Expression of *CYCLIND6;1* in *shr2* mutants with NP, peptide treatments, or DEX control treatment. (Scale bar, 25 µm in *A*, *C*, and *E*.)

To test this association further, we sought to identify which of the 32 *Arabidopsis* CLE peptides could act as ligands for BAM1/2 in formative ground tissue divisions. *CLE* genes are not well represented on microarrays and often lowly expressed. Therefore, we used a validated stem cell niche-specific transcriptional profiling set generated from sorted and RNA sequenced root cell types to identify *CLE* genes expressed in, or near, CEI cells (25). We identified several *CLE* genes expressed in or near the CEI region (*SI Appendix*, Fig. S3*A* and Table S1). We confirmed these expression patterns using previously published native *CLE* promoter transcriptional *GUS* reporters (26) (*SI Appendix*, Fig. S3*B*). CLE propeptides are processed proteolytically to release active dodecapeptides (CLEp) (27), which are secreted into the extracellular space, where they bind and activate BAM/CLV1 family receptors (28, 29). We predicted that if CEI region-expressed *CLE* genes encoded relevant BAM1/2 ligands, and contributed to spatial control of formative divisions, then exogenous application of the corresponding CLEp might be sufficient to activate *CYCD6;1* expression and ectopic formative cell divisions. Exogenous application of dodecapeptides corresponding to a subset of root stem cell niche-expressed *CLE* genes was indeed sufficient to up-regulate *CYCD6;1* expression in roots (Fig. 3*A* and *SI Appendix*, Table S1). CLE16p and CLE13p were the most effective at up-regulating *CYCD6;1*, with ectopic expression expanding proximally throughout the endodermal layer. Notably, CLE peptide treatment did not alter *DR5::GFP* auxin transcriptional reporter expression, demonstrating signaling output specificity (*SI Appendix*, Fig. S3*C*). Consistent with the up-regulation of *CYCD6;1*, we determined that exogenous CLE13p and CLE16p also triggered ectopic cell divisions in ground tissue in proximal root regions, using the ground tissue marker J0571, with entire extra layers of ground tissue being formed in some cases (Fig. 3*B*). CLE16p treatment of *EN7p::H2B-YFP* reporter lines revealed that ectopic ground tissue divisions were asymmetric formative divisions, similar to wild-type CEID divisions, with endodermal identity being restricted to new inner cell layers following ectopic divisions (Fig. 3*B*). CLE16p treatment failed to increase ground tissue layer number in *bam1/2/3* triple mutants, indicating that BAM receptors are required for CLE16p-induced cell divisions (Fig. 3*C*). To further confirm the biological relevance of the peptide assays, we screened a collection of CRISPR-generated *cle* null mutants and found significantly reduced CEI/CEID divisions, comparable to *bam1* single mutants, in three independent *cle16* mutant alleles (30) (Fig. 3*D* and *SI Appendix*, Fig. S3 *D* and *E*), which was rescued with *CLE16p::CLE16* in *cle16-4* (Fig. 3*D*). Accordingly, CLE13p and CLE16p bound to the purified BAM1 extracellular domain in vitro with high affinity, with dissociation constants of 10 nM and 6.9 nM, respectively (Fig. 3*E*), comparable to previously reported CLE9p-BAM1 interactions (31). Exogenous CLE16p did not alter *SHR* or *SCR* expression patterns, or SHR protein localization, consistent with the lack of *SHR*/*SCR* expression changes in *bam1/2/3* mutants (*SI Appendix*, Fig. S3 *F* and *G*). Collectively, these results show that CLE16p signals through BAM receptors, redundantly with other CLE peptides, and demonstrates that CLE-BAM signaling is necessary and sufficient to activate *CYCD6;1* expression and formative cell divisions.

**Fig. 4.**
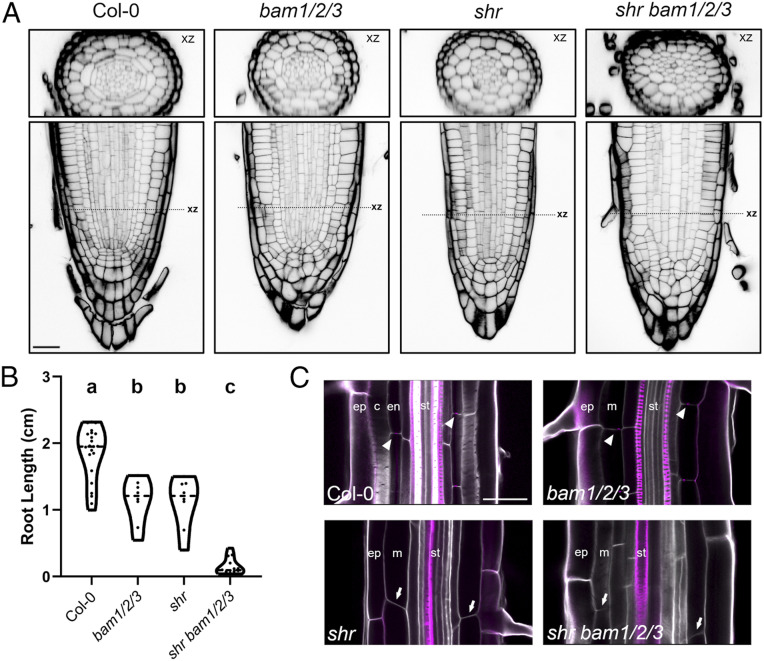
BAM1/2 and SHR pathways impact cell division patterns throughout the stem cell niche. (*A*) Representative longitudinal (xy) and radial (xz) images of Col-0, *bam1/2/3*, *shr*, and *shr bam1/2/3* seedling phenotypes at 7 dag showing extensive root disorganization throughout where the QC, ground tissue, and stele are found. (*B*) The quadruple mutant displays an enhanced root elongation defect in 7-d-old seedlings. Distributions were compared using a Brown–Forsythe and Welch ANOVA (*P* < 0.01). (*C*) Casparian strip formation was observed by basic fuschin staining in Col-0 and *bam1/2/3* but was absent in both *shr* and *shr bam1/2/3* (arrowheads, Casparian strip; arrows, no Casparian strip). Ep, epidermis; c, cortex; en, endodermis; st, stele. (Scale bar, 25 µm in *A* and *C*.)

*CYCD6;1* is a direct SHR/SCR target and reporter expression in CLEp-treated plants was up-regulated in the endodermal tissue layer, where nuclear SHR/SCR complexes accumulate in wild-type plants, and was not seen in stele or epidermal cells. Given this, and the congruence of *bam* and *shr-scr* mutant plant phenotypes, we speculated that SHR might be necessary for BAM1/2-CLE16p signaling in formative divisions. Indeed, CLE16p and CLE13p treatment failed to activate *CYCD6:1* expression in *shr* null mutant plants carrying a *SHRp::SHR-GR* transgene in the absence of dexamethasone (8) (Fig. 3*F*), demonstrating that nuclear *SHR* is necessary for downstream BAM1/2-*CYCD6;1* signaling outputs. To test the relationship further, we generated *shr bam1/2/3* quadruple null mutants. Confocal imaging of *shr bam1/2/3* quadruple null mutants surprisingly revealed extremely disorganized cell division patterns throughout the stem cell niche which were not seen in either parental plant. These defects were highly variable across plants precluding simple quantification (Fig. 4*A*). Additionally, *bam1/2/3* mutants and *shr* additively impaired root growth, which could be either direct or indirect, or due to the disorganized root patterning (Fig. 4*B*). Consistent with our data on marker gene expression, *bam1/2/3* mutants formed a Casparian strip, which was abolished in *shr bam1/2/3* quadruple null mutants (Fig. 4*C*). Collectively, our data point to an unappreciated congruence between SHR and BAM1/2 signaling in the control of root cell divisions, independent of SHR-mediated cell identity. While little is known about cell cycle control in other root tissues, at minimum our data demonstrate that during formative ground tissue divisions these pathways converge to promote *CYCLIND6;1* expression. It will be of interest to see if a similar regulatory logic occurs in other root divisions.

## Discussion

Our work establishes CLE-BAM signaling as a key regulator of *CYCD6;1* expression and formative root and hypocotyl ground tissue cell divisions. As such, animals and plants independently evolved receptor kinase-mediated control of transcriptional cell cycle regulation during development. BAM1/2 are required for correct SHR-mediated *CYCD6;1* expression and *SHR* is in turn required for ectopic CLE peptide-mediated formative divisions and *CYCD6;1* expression. Although the relationship between BAM1/2 signaling and SHR is not clear, it is tempting to speculate that BAM signaling could regulate local SHR/SCR transcriptional function at the distal root tip, perhaps directly as a downstream signaling target. In such a model, root tip-expressed CLE peptides would trigger BAM1/2 signaling which might regulate SHR/SCR function by phosphorylation of interacting target proteins, to specifically influence formative cell divisions, without impacting root layer identity. This interpretation would explain the congruence of loss-of-function phenotypes and the mutual dependence of SHR and BAM1/2 signaling. Alternatively, BAM1/2 signaling might be permissive for SHR-mediated *CYCD6;1* expression and division by impacting a shared process, which might explain the extensive root division defects in the quadruple *shr bam1/2/3* mutant. These two models are not mutually exclusive and point to the existence of unappreciated overlapping for these pathways beyond CEI divisions. While little is known about cell cycle control outside of CEI division in roots, it is tempting to speculate a more general role for both pathways in cell cycle gene expression in other cell types. Interestingly, when *CLE16* is not produced, distal formative CEI divisions are compromised and when CLE16p is exogenously applied to wild-type plants, formative cell divisions increase but also expand proximally. How the broader SHR/SCR complex promotes formative divisions specifically at distal root tips in CEI cells is unknown, but our work supports a role for CLE-BAM signaling in this mechanism. While it is not technically feasible to determine native CLE peptide concentration gradients in vivo, it is possible that distal accumulation of CLE16p and other CLEp contribute to spatially restricted CEI divisions. The downstream signaling pathways of BAM receptors are unknown, and there are several potential candidate SHR/SCR interactors, including the RBR1 cell cycle regulator, which is conserved between animals and plants and a known target of animal mitogenic pathways (32). While technically challenging, given the small number of cells relevant to the phenotype and the necessity to select homozygous *bam1/2* mutants in segregating populations at early seedling stages, it will be of interest to see if the evolutionarily distinct extracellular signaling pathways converge on conserved phosphorylation targets between animals and plants. The control of cell division by CLE signaling predates the evolution of roots (33, 34) and SHR-like regulators are conserved in basal plants. It will be of interest to see if there is a potential ancient origin for this connection in the plant kingdom.

## Materials and Methods

### Plant Lines.

Mutant seed stocks used in this study are summarized in *SI Appendix*, Table S2. The mutant fluorescent reporter lines for root cell identity were generated by crossing *bam1/2/3* (17) to the ground tissue marker lines for the endodermis (*EN7p::H2B-YFP*), cortex (*Co2p::H2B-YFP*), and CEI (*CYCD6;1p::GFP*) (8, 35). The lines *SCRp::SCR-GFP*, *SHRp::SHR-GFP*, *SCRp::erGFP*, *CASP1p::2xmCherry*, and *SHRp::SHR-GR shr-2* (3, 6, 8, 21, 36) were also introgressed into *bam1/2/3*. We took advantage of the unique, unrelated cotyledon phenotype in *bam1/2/3* triple mutant plants to help select mutants at critical early stages in experiments. *BAM1p::BAM1-GFP* (17) was introduced into *bam1 bam2/+ bam3* plants by floral dip and isolated in subsequent generations. Genotyping primers used are listed in *SI Appendix*, Table S3. *CLE13p::GUS* and *CLE16p::GUS* lines (26) were obtained from The Arabidopsis Information Resource. Additional *cle16* alleles (*cle16-2* and *cle16-3*) are previously described (30).

### Growth Conditions and Peptide and Chemical Treatments.

Seeds were surface sterilized with 70% ethanol and 0.1% Triton X-100 for 10 min, rinsed three times with 70% ethanol, and plated onto 0.5× Murashige and Skoog (MS) (pH 5.7) (Research Products International) with 8 g/L Phytoagar (Research Products International) and stratified at 4 °C for 2 d. Following stratification, seeds were germinated in a continuous light growth chamber at 22 °C to 24 °C. For peptide-treated plants, sterilized seeds were stratified and germinated on 0.5× MS plates. At 3 d after germination (dag), seedlings were transferred to either 0.5× MS plates or 0.5× MS plates supplemented with 0.1 μM CLEp for 48 h. Roots were imaged 5 dag as described in the figure legends. All synthetic peptides (>90% purity, Biomatik) (*SI Appendix*, Table S1) were dissolved in sterile dH_2_O as recommended. For treatment with dexamethasone (Dex; Sigma), seedlings were stratified and germinated on 0.5× MS plates supplemented with 10 μM Dex for 3 d before imaging.

### Generation of *cle16-4* Using CRISPR-Cas9.

The CRISPR-Cas9 *pCUT* vector (37) containing a sgRNA targeting *CLE16* was introduced into Col-0 by floral dip. The target sequence (5′-3′) of TTG​TTC​CAG​AAA​AAG​AAG​A had no predictable off-targets and was used as a source for dCAP marker screening (*SI Appendix*, Table S3) in subsequent generations. Transgene-free *cle16-4* was isolated in the T3 generation as a single A-bp insertion at bp 45, resulting in a frameshift after codon 8 and thereby, a stop codon at codon 81, fully truncating the CLE16 protein prior to the CLE domain.

### Genetic Complementation of *cle16-4.*

*CLE16* (At2g01505) and surrounding promoter regions (2.5 kB upstream and 0.35 kB downstream) were amplified from Col-0 genomic DNA. The fragment was cloned into the entry vector, pDONR207 and the binary vector, GWB501 (Addgene, plasmid #74843) using standard Gateway cloning methods (Invitrogen). The transgene was introduced into *cle16-4* by floral dip and genotyped T2 plants were used for complementation analysis.

### Confocal Microscopy and Histological Sectioning.

Laser scanning confocal microscopy of roots was performed using either a C-Apochromat 40×/1.20 W Korr objective on a Zeiss laser scanning microscopy (LSM) 710 or an EC Plan-Neofluar 40×/1.30 oil differential interference contrast (DIC) M27 objective on a Zeiss LSM 880. Roots were examined by staining with 10 μM propidium iodide (PI) (Sigma-Aldrich). For images in Figs. 2*B* and 4 *A* and *C* and *SI Appendix*, Figs. S1*D* and S2*B*, seedlings were fixed in 4% paraformaldehyde in phosphate-buffered saline (PBS) for 45 min, rinsed in PBS, and cleared in ClearSee solution (38) overnight. Fixed seedlings were incubated in 0.2% Calcofluor white (Sigma-Aldrich) for 30 min and transferred to fresh ClearSee solution 2 to 24 h before imaging. Laser line excitations and emissions are as follows: Calcofluor white (405 nm; 410 to 551 nm), GFP (488 nm; 492 to 551 nm), YFP (514 nm; 519 to 564 nm), mCherry (561 nm; 566 to 606 nm), and PI (561 nm; 566 to 682 nm). Histological sectioning of Col-0 and *bam1/2/3* roots 6 dag were prepared as previously described (39).

### GUS Staining for Detection of CLE Gene Expression.

*CLE13p::GUS* and *CLE16p::GUS* transgenic lines are previously described (26). Gus staining was performed as published (40) with few alterations. In brief, seedlings were harvested at 5 dag and fixed in 90% acetone at −20 °C for 30 min. Following fixation, seedling tissue was rinsed in 100 mM phosphate buffer (pH 7.2) and incubated with GUS staining solution at 37 °C for 2 d. Tissue was rinsed again in phosphate buffer, moved to 70% ethanol for clearing and preservation, and stored at 4 °C until imaging. Images were taken on a Nikon Eclipse 80i compound microscope using DIC optics.

### Root Cell Sorting and RNA-Seq Transcriptional Profiling.

The details of the cell sorting and transcriptional profiling are recently published in ref. 25.

### Protein Expression and Purification.

The coding sequence of BAM1 (residues 20 to 637) was synthesized (GeneArt) with codons optimized for expression in *Trichoplusia ni* and cloned in a modified *pFastBac* vector (Geneva Biotech), harboring the *Drosophila* BiP secretion signal peptide and a TEV (tobacco etch virus protease) cleavable C-terminal StrepII, 10× His tag and a noncleavable Avi-tag (41, 42). *T. ni* (strain Tnao38) (43) cells were infected with a multiplicity of infection (MOI) of 1 at a density of 2 × 10^6^ cells mL^−1^ and incubated 26 h at 28 °C and 48 h at 22 °C. The secreted protein was purified from the supernatant by Ni^2+^ (HisTrap Excel; GE Healthcare; equilibrated in 50 mM KP_i_ pH 7.6, 250 mM NaCl, 1 mM 2-mercaptoethanol) and StrepII (Strep-Tactin XT Superflow high affinity chromatography: IBA; equilibrated in 20 mM Tris pH 8.0, 250 mM NaCl, 1 mM ethylenediaminetetraacetic acid) affinity chromatography. The tag was cleaved with His-tagged TEV protease at 4 °C overnight and removed by Ni^2+^ affinity chromatography. Proteins were then further purified by size-exclusion chromatography on a Superdex 200 increase 10/300 GL column (GE Healthcare), equilibrated in 20 mM sodium citrate pH 5.0, 250 mM NaCl.

### Protein Biotinylation.

BAM1 protein (20 µM) was biotinylated with biotin ligase BirA (2 µM) (42) for 1 h at 25 °C, in a volume of 200 µL; 25 mM Tris pH 8, 150 mM NaCl, 5 mM MgCl2, 2 mM 2-mercaptoethanol, 0.15 mM biotin, 2 mM ATP, and followed by size-exclusion chromatography to purify the biotinylated protein.

### Grating-Coupled Interferometry.

Grating-coupled interferometry (GCI) experiments were derived by the Creoptix WAVE system (Creoptix). All experiments were performed on 4PCP WAVE chips (quasiplanar polycarboxylate surface; Creoptix). Borate buffer (100 mM sodium borate pH 9.0, 1 M NaCl; Xantec) was used for chip conditioning and streptavidin (Sigma) was immobilized on the chip surface with standard amine coupling; 7 min activation [1:1 mix of 400 mM *N*-(3-dimethylaminopropyl)-*N′*-ethylcarbodiimide hydrochloride and 100 mM *N*-hydroxysuccinimide] (Xantec), followed by injection of streptavidin (30 µg mL^−1^) in 10 mM sodium acetate pH 5.0 (Sigma) until the desired density was reached, passivation of the surface (0.5% bovine serum albumin [Roche] in 10 mM sodium acetate pH 5.0) and final quenching with 1 M ethanolamine pH 8.0 for 7 min (Xantec). Then, biotinylated BAM1 (80 µg mL^−1^) was captured on the chip surface. All kinetic analyses were performed at 25 °C with a 1:2 dilution series from 100 nM for CLE9 or 10 µM for the other peptides in 20 mM citrate pH 5.0, 250 mM NaCl, 0.01% Tween 20. Blank injections were used for double referencing and a dimethyl sulfoxide (DMSO) calibration curve for bulk correction. Analysis and correction of the obtained data were performed using the Creoptix WAVE control software (correction applied: X and Y offset; DMSO calibration; double referencing). Mass transport binding models with bulk correction were used to fit all experiments.

## Supplementary Material

Supplementary File

## Data Availability

All study data are included in the article and *SI Appendix*.
