# Supplementary material for: BAM1/2 receptor kinase signaling drives CLE peptide-mediated formative cell divisions in Arabidopsis roots
Source: Proc Natl Acad Sci U S A. 2020 Dec 7;117(51):32750–6. doi: 10.1073/pnas.2018565117 (PMC7768756; doi:10.1073/pnas.2018565117)
Supplement: Supplementary File [file pnas.2018565117.sapp.pdf]

PNAS
www.pnas.org

Supplementary Information for

BAM1/2 receptor kinase signaling drives CLE-peptide mediated formative cell divisions in Arabidopsis roots

Ashley D. Crook[a,1], Andrew C. Willoughby[a,1], Ora Hazak[b,2], Satohiro Okuda[c,3], Kylie R. VanDerMolen[a], Cara L. Soyars[a], Pietro Cattaneo[b], Natalie M. Clark[d], Rosangela Sozzani[d], Michael Hothorn[c], Christian S. Hardtke[b], and Zachary L. Nimchuk[a,e,4].

Corresponding author: Zachary L. Nimchuk
Email: zackn@email.unc.edu

This PDF file includes:

Figures S1 to S3
Tables S1 to S3
SI References

www.pnas.org/cgi/doi/10.1073/pnas.2018565117

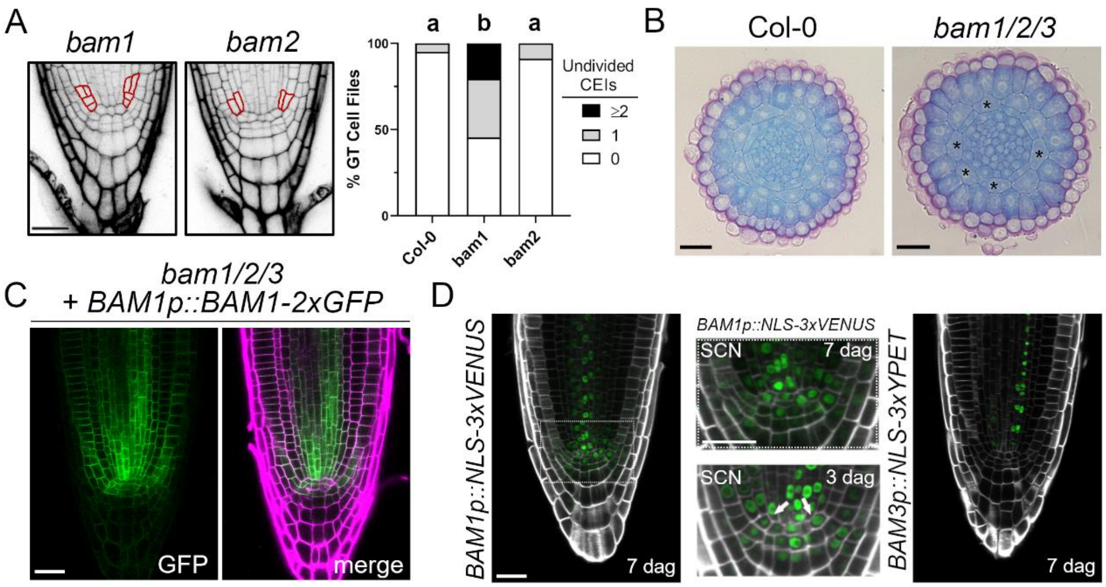

Fig. S1. BAM1/2 receptors are required for formative root cell divisions. (A) Quantification of undivided CEI cells in *bam1* and *bam2* single mutants (5 dag; n=81, Col-0; n= 56, *bam1*; n= 48, *bam2*). Undivided CEI cells and the first set of divisions are outlined in red. Distributions were compared using a Kruskal-Wallis nonparametric test. (B) Cross-sections of Col-0 and *bam1/2/3* roots at 6 dag. Asterisks represent a GT cell file with no formative division. (C) The division defects of a *bam1/2/3* mutant are rescued by the *BAM1p*::*BAM1-2xGFP* transgene. (D) The promoter of *BAM1* is active throughout the stem cell niche (SCN; boxed area), ground tissue (including CEIs at 3 dag, arrows), and stele in roots at 3 and 7 dag. (D) *BAM3* expression is restricted mainly to phloem lineage cells (D). Scale bars = 25 uM (A-D).

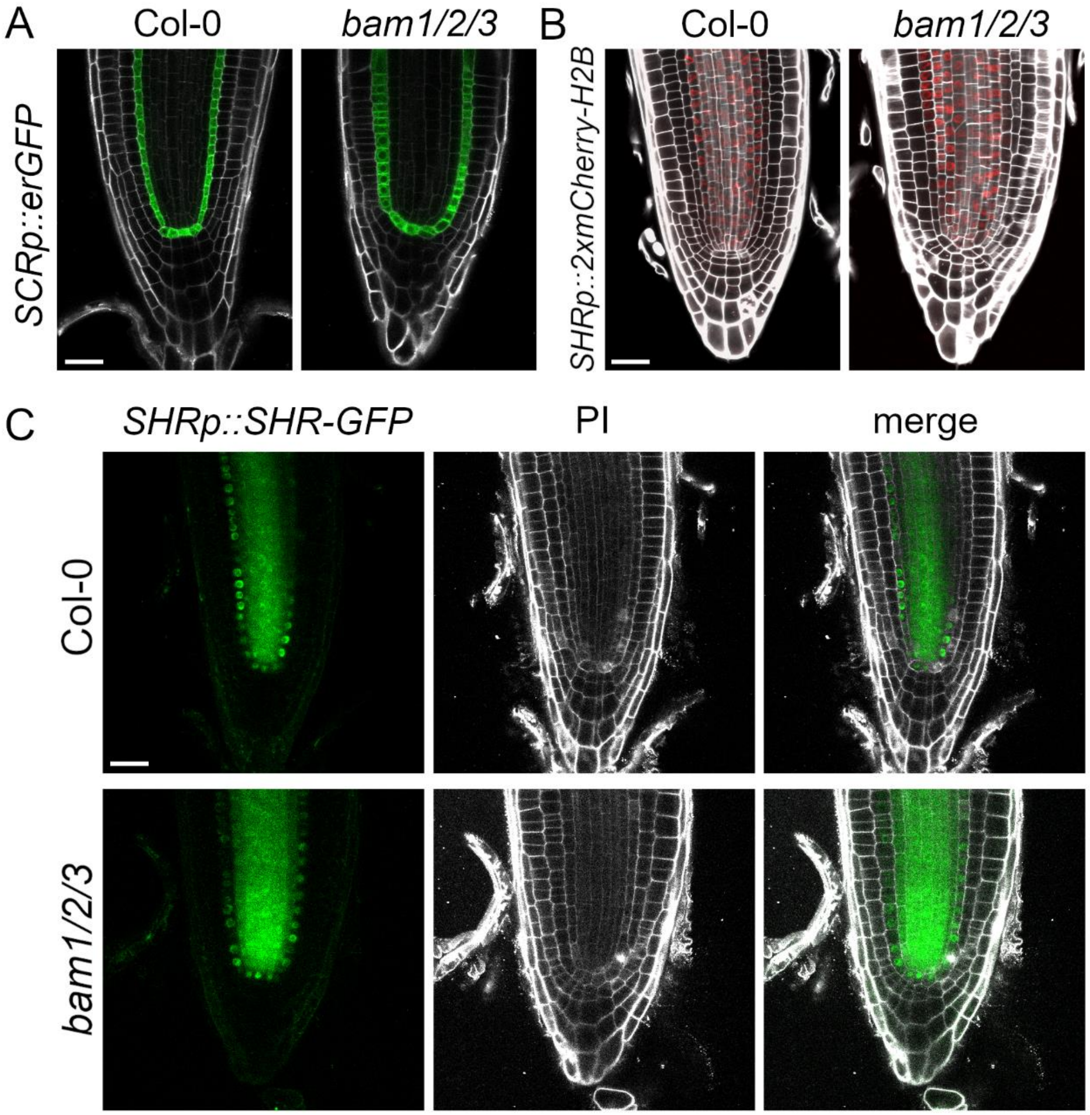

Fig. S2. BAM1/2 do not regulate *SHR* or *SCR* expression or protein dynamics. (A-B) Expression patterns of *SCR* (A; 7 dag) and *SHR* (B; 4 dag) were unchanged in *bam1/2/3* roots when compared to Col-0 roots. (C) SHR-GFP was localized in the stele of Col-0 and *bam1/2/3* and the subsequent trafficking into the ground tissue and nuclear sequestration was observed. Scale bar = 25 µm (A-C).

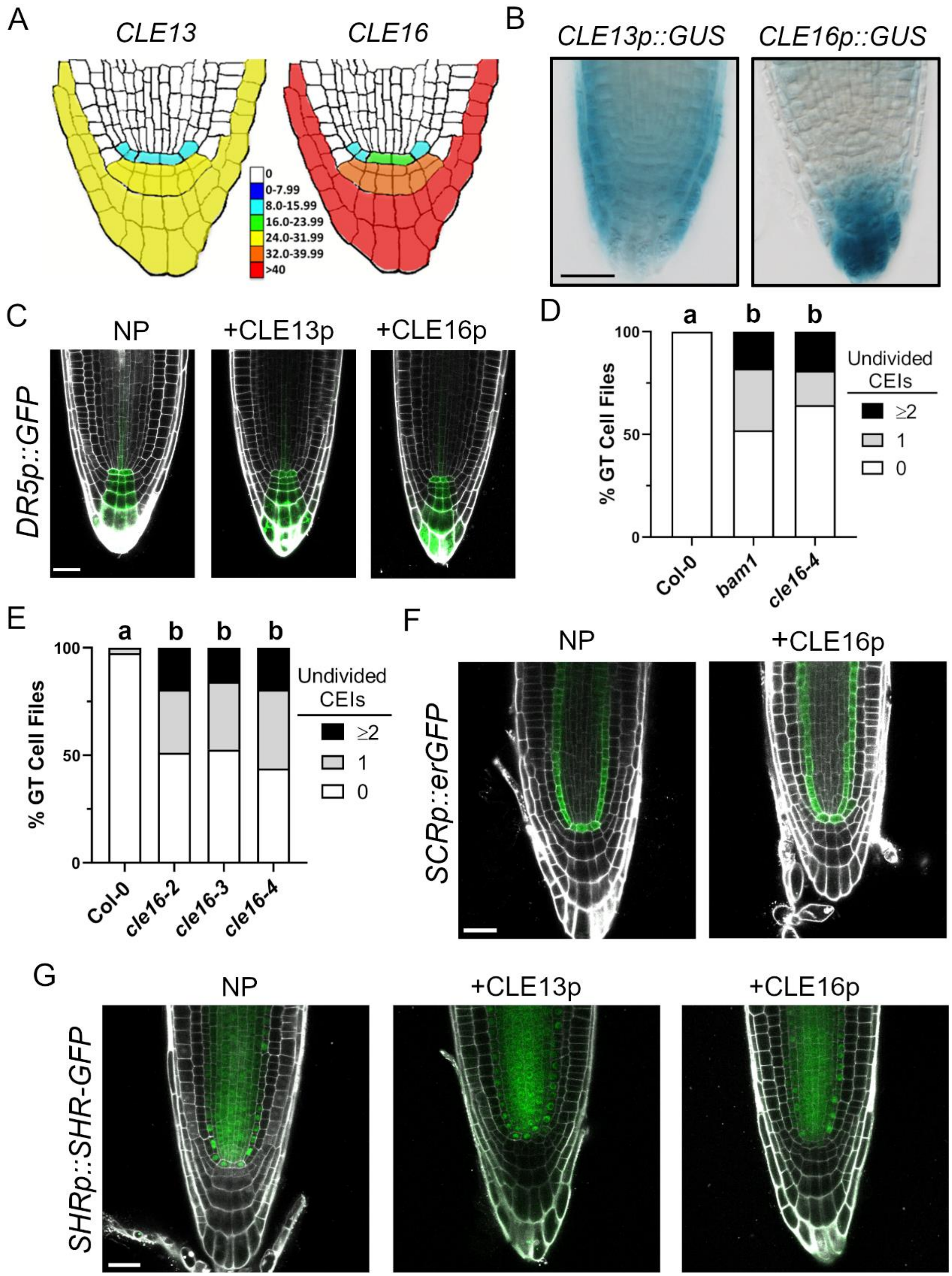

Fig. S3. Root enriched CLE genes regulate formative cell divisions. (A) Expression profiles of *CLE13* and *CLE16* by cell-type specific RNA-seq (1) (heatmap represents relative transcript abundance) and (B) *promoter::GUS* fusions in the root meristem. *CLE13* is largely expressed

throughout the stem cell niche, with *CLE16* expression is most prominent in columella and lateral root cap cells. (C) The expression pattern of *DR5p::GFP* does not change in response to peptide treatment in plants at 3 dag. (D) Undivided CEI quantification in the GT cell files of *bam1* and *cle16-4* (n= 32, Col-0; n= 50, *bam1*; n= 42, *cle16-4*) show similar division defects. (E) Three independent mutant alleles of *CLE16* display congruent phenotypes (n= 40, Col-0; n= 41, *cle16-2*; n= 38, *cle16-3*; n= 41, *cle16-4*). Distributions were compared using a Kruskal-Wallis nonparametric test. (F-G) Confocal imaging of roots expressing *SCRp::erGFP* (F) and *SHRp::SHR-GFP* (G) did not show altered patterns when treated with peptides. Scale bar = 25 µm (B, C, F, and G).

Table S1. Locus ID, sequence, and *CYCLIND6;1p::GFP* response of synthesized root expressed CLE peptides.

CLEp	Locus ID	Sequence	# AA	Upregulation of *CYCD6* observed?
CLE1p	At1g73165	RLSPGGPDPRHH	12	No
CLE2p	At4g18510	RLSPGGPDPQHH	12	No
CLE12p	At1g68795	RRVPSGPNPLHH	12	Yes
CLE13p	At1g73965	RLVPSGPNPLHH	12	Yes
CLE14p	At1g63245	RLVPKGPNPLHN	12	n.d.
CLE16p	At2g01505	RLVHTGPNPLHN	12	Yes

n.d. not determined

Table S2. Locus ID and allele information for genotypes used in the study.

Genotype	Locus ID	Mutant allele
BAM1	At5g65700	*bam1-4*, SALK_107290
BAM2	At3g49670	*bam2-4*, SAIL_1053_E09
BAM3	At4g20270	*bam3-2*, SALK_044433
		SALK_004121
SHR	At4g37650	*shr-2*, CS2972
SCR	At3g54220	*scr-3*, CS3997
CLE16	At2g01505	*cle16-2*
		cle16-3
		cle16-4

Table S3. Primer sequences used for cloning and genotyping.

Genotype		Primer sequence (5'-3')	Notes
BAM1	F	ccggtactctttccccagatgtttctcatttacgtc	
	R	cttattggaagagagatcgacgagatttagtttacc	
bam1-4	F	cttattggaagagagatcgacgagatttagtttacc	
	R	attttgccgatttcggaac	T-DNA LB primer (LB1.1a)
BAM2	F	tatggttcgctttggtattg	
	R	gttagctcgttaccggaaacc	
bam2-4	F	gaagtccagctgccagaaac	detects BASTA^{r}
	R	gcaccatcgtcaaccactac	
BAM3	F	ggtgaagataacacaacccccttagccgcttccaacg	
	R	ccggtactctttccccagatgtttctcatttacgtc	
bam3-2/ SALK_004121	F	ccggtactctttccccagatgtttctcatttacgtc	
	R	attttgccgatttcggaac	T-DNA LB primer (LB1.1a)
SHR/shr-2	F	actcctccgtccttcgactt	*shr-2* (large indel)
	R	tctgtggctgcagctgttac	
SCR/*scr-3*	F	tcacgggacttggtacttcc	CAPs; MaeII site is introduced in *scr-3*
	R	cttctcgatggtcctccaac	
CLE16/*cle16-2*	F	gaatccaaaacctgctctgc	MspI site at +59 is altered in *cle16-2* (+C)
	R	cgaaggagcagtcaacacct	
CLE16/*cle16-3*	F	gaatccaaaacctgctctgc	MspI site at +59 is altered in *cle16-3* (-G)
	R	cgaaggagcagtcaacacct	
CLE16/*cle16-4*	F	caaatcaaacagccatggaagcttgttccagaaccaga	dCAPs; BseL1 site at +45 is altered in *cle16-4* (+A)
	R	cttggagagagaccagacac	
CLE16p::CLE16	F	GGGGACAAGTTTGTACAAAAAAGCAGGCTTCACCtatgcacttaaagtgtggtaacactg	attB1-*CLE16* 5'promoter
	R	GGGGACCACTTTGTACAAGAAAGCTGGGTGgatcttcgaagaaatccatgcattcg	attB2-*CLE16* 3' UTR

SI References

1. Clark, N., *et al.* Stem-cell-ubiquitous genes spatiotemporally coordinate division through regulation of stem-cell-specific gene networks. Nat. Commun. **10**, 5574 (2019).
